# Perception regarding the causes of schizophrenia and associated factors among Feresbet district residents: a community based study

**DOI:** 10.1186/s12889-019-6678-4

**Published:** 2019-03-25

**Authors:** Zelalem Belayneh, Dessie Abebaw, Tadele Amare, Kibrom Haile, Zegeye Abebe

**Affiliations:** 1 Department of Psychiatry, College of Health and Medical Sciences, Dilla University, Dilla, Ethiopia; 2 Department of Epidemiology and Biostatistics, Institute of Public Health, College of Medicine and Health Sciences, University of Gondar, Gondar, Ethiopia; 3 Department of Psychiatry, School of Medicine, College of Medicine and Health Sciences, University of Gondar, Gondar, Ethiopia; 4 Amanuel Mental Specialized Hospital, Research and Training Directorate Office, Addis Ababa, Ethiopia; 5 Department of Human Nutrition, Institute of Public Health, College of Medicine and Health Sciences, University of Gondar, Gondar, Ethiopia

**Keywords:** Schizophrenia, Feresbet, Perception, Causes of schizophrenia, Causal model questionnaire for schizophrenia, Community perception

## Abstract

**Background:**

A wide variety of beliefs exist in the public towards schizophrenia. Community perception about the causes of schizophrenia can affect the way of seeking help, treatment outcomes, and community integration of individuals with schizophrenia. Therefore, assessing the community perception and associated factors about the causes of schizophrenia is vital.

**Method:**

A cross-sectional study was conducted among Feresbet district residents through a multi- stage sampling technique. A causal model questionnaire for schizophrenia (CMQS) was used to assess the perceived causes of schizophrenia. The collected data were explored to SPSS version 20 for analysis. Bi-variable and multi variable logistic regression were computed to identify factors associated with the traditional perception about the causes of schizophrenia and the level of significance were determined at a *P*- value < 0.05 with 95% CI.

**Results:**

Out of the total study participants, about 73.7% had the traditional perception regarding the causes of schizophrenia. According to multivariate analysis, female sex, no formal education, age ≥ 25 years, living in the extended family system, and being unemployed had a significant association with the traditional perception of the cause of schizophrenia.

**Conclusions:**

The traditional perception of the cause of schizophrenia is higher than the bio-psycho-social view. Female sex, no formal education, age ≥ 25 years, living in an extended families and unemployed had a significant association with the traditional perception of the causes of schizophrenia. Therefore, giving special attention to females, uneducated and unemployed individual is crucial. In addition, older age and individuals living in extended family system need attention regarding the possible causes of schizophrenia.

**Electronic supplementary material:**

The online version of this article (10.1186/s12889-019-6678-4) contains supplementary material, which is available to authorized users.

## Background

Schizophrenia is a severe mental disorder characterized by fundamental disturbances in thinking, perception, behaviors, and emotions. It affects approximately 1% of the world’s population and ranked as the 8th leading cause of years lived with disability (DALYs) worldwide [1–3].

Although the biopsychosocial model is the prevailing wisdom at the current time, there are also wide varieties of traditional beliefs (demon possessions, bewitchments, evil spirit, evil eye, God’s will, magic, curse and punishment for sins and others) exists in the public regarding the causes of schizophrenia [4–6]. However, all most all the communities in the world explained that more than one single reason could be the possible causes of schizophrenia [7].

Traditional perspectives are observed both in the developing and developed nations, but developed nations have a better biopsycho-social view about the causes of schizophrenia [8–10].

A survey done in Pakistan reported that numerous participants shared traditional perceptions including God’s will (32.3%), superstitious ideas (33.1%), loneliness (24.8%) and unemployment (19.3%) while only 30% of the participants attributed “mental illness” as the possible cause of schizophrenia [11]. Similarly, in Ghana, witchcraft/evil spirits and divine punishment were endorsed as causes of schizophrenia by 94 and 66% of the community members, respectively [12].

The context of beliefs and perception held by the patients, their family members and the community with respect to the causes of schizophrenia can affect the early detection, help-seeking behavior, adherence to treatment and the way in which individuals with schizophrenia integrate into the community [13, 14].

A biopsycho-social view about the causes of schizophrenia is associated with a more tolerant, less stigmatizing, and best professional help-seeking attitude. On the other hand, supernatural perception may result in stigma, treatment delay, poor drug adherence [15], poor treatment outcome, functional deterioration, and negatively affects the prognosis of the illness [16–18].

In Ethiopia, little information is available about the magnitude of schizophrenia and its perceived causes [19, 20]. Therefore, this study was aimed to assess the perceived causes and associated factors of schizophrenia among Feresbet district residents, in the northwest part of Ethiopia.

## Methods

### Study design, period and setting

A community-based cross-sectional study was conducted at the Feresbet district from December 1st/2016 to February 1st^/^2017. The district is found in Amhara regional state of Ethiopia. The district has one primary hospital, one health center and three health posts serving for about 15, 342 population. But there is no mental health professional in any of these health institutions.

### Sample size determination and sampling procedure

The sample size was calculated by using Epi-info software version 3.5 by considering the following assumptions: 95% confidence interval, 80% power, 60.7% proportion of factor of traditional perception of schizophrenia (living in an extended family system), odds ratio of 2, and design effect of 2. Finally, by adding a 10% non-response rate, a sample size of 964 was found.

A multistage stratified sampling followed by systematic random sampling technique was employed to select the study participants. Initially, one kebele (the smallest administrative unit in Ethiopia) from the three kebeles was randomly selected. The total number of households of the selected kebele was found from the town administration. Then, the sampling interval (K) was determined by dividing the total of number of households in the selected kebele to the sample size to be drawn from that kebele. To determine the starting point, a lottery method was used to select one household between one and K. Subsequently, K value was added to the calculated sample. Individual adults who were a permanent resident of the town (who resides at least for six months) were invited for participation.

### Data collection

A face to face interviewer-administered questionnaire was used to collect the data. The questionnaire had five parts consisting of the socio-demographic characteristic, the case vignette, the Causal Models Questionnaire for Schizophrenia (CMQS), sources of information and exposure related questions**.**

The CMQS has 36 items to be asked whether individuals perceived each item as a possible cause of schizophrenia or not. It has been used in different studies to assess the perceived causes of schizophrenia with good reliability [13, 21, 22].

A case vignette was developed by the investigators based on the proper DSM-V diagnostic criteria of schizophrenia and commented by senior psychiatrists in Amanuel Mental Specialized Hospital (AMSH) (Additional file 1). Finally, the questionnaire was translated into Amharic (the mother tongue of the study participants) and back to English to check its consistency. Two days training were given for data collectors and supervisors. The pretest was done on 5% of the sample at Finotselam town residents before the actual data collection. The data collectors obtained written consent from each respondent after a brief explanation about the scope and objectives of the study. Then, the unlabeled case vignette descriptions of schizophrenia were read once to each participant followed by questions “what do you label in your local context and what you explain about the causes of the girl’s condition in the case vignette from the 36 items of CMQS”. Participants were also asked what they perceive as a single most important cause for the girl’s condition in the case vignette from the CMQS items. The vignette was unlabeled, and the diagnosis was not revealed by the interviewer throughout. Respondents were asked to label it and the illness label employed by the respondents was used to measure their perception. Then, the response of each respondent was categorized into two mutually exclusive themes (traditional and bio-psychosocial causes).

Finally, respondents were asked regarding their source of information and exposure history with regard to someone with similar problems as described in the vignette.

### Operational definition

Kebele: It is the smallest administrative unit in Ethiopia consists of 5000 people.

Traditional view of schizophrenia is defined as the individual’s perception about the cause of schizophrenia as it is due to demonic possessions, bewitchments, evil spirit, evil eye, God’s will, magic, curse and punishment for sins.

Bio-psychosocial view of schizophrenia is defined as the individual’s perception about the cause of schizophrenia as it is due to biological, psychological and social problems.

### Data management and analysis

First, the collected data were manually checked for its completeness and consistency. Then, it was entered the computer using EP- info version 3.5 software and exported SPSS version 20 for analysis.

Descriptive statistics were used to explain the study participants in relation to the study variables and the results were presented using tables and text. Both bi-variable and multi-variable logistic regression analyses were used to identify factors associated with the traditional perception about the cause of schizophrenia. Variables with a *P*-value of less than 0.2 in the bi-variable analysis were entered into multi-variable logistic regression models. Variables with a P-value of less than < 0.05 levels in the multi-variable logistic regression were considered as statistically significant. The strength of association was estimated using odds ratio (OR) with 95% confidence interval (CI).

## Results

### Socio-demographic characteristics of the respondents

A total of 952 individuals participated in this study with a response rate of 98.7%. The mean (±SD) age of respondents was 34 (±11.7) years. More than half, 545 (57.2%), of the respondents were females. All the respondents were Amhara by ethnicity and Orthodox Christian in their religion.

Regarding their marital status, 347 (36.4%) of the participants were married and lived together. About 209 (26.8%), 215 (22.6%), and 727(81%) of participants were completed secondary school and 727(81.0%) were living in a nuclear family system **(**Table 1**).**

**Table 1:** Socio-demographic characteristics of Feresbet district residents, December 1st, 2016 to February 1st, 2017 (*n* = 952)

Variables	Categories	Frequency	Percentage
Age in years	18–24	277	29.1
	25–44	343	36.0
	45–64	186	19.5
	> = 65	146	15.3
Sex	Male	407	42.8
	Female	545	57.2
Marital status	Married	327	34.3
	Single	347	36.4
	Separated	93	9.8
	Divorced	79	8.3
	Widowed	106	11.1
Educational level	Unable to read and write	164	17.2
	Able to read and write	139	14.6
	Primary	185	19.4
	Secondary	255	26.8
	Diploma and above	209	22.0
Occupation	Government employed	215	22.6
	Unemployed	164	17.2
	Private business	184	19.3
	Daily laborer	49	5.1
	Homemakers	194	20.4
	Student	110	11.6
	Others^a^	36	3.8
Family system	Nuclear	771	81.0
	Extended	181	19.0

^a^others = local beer making, house servant etc

### Sources of information and exposure to people with schizophrenia related problems

Around 702 (73.7%) of the participants heard information related to problems as described in the case vignette from different sources (47.70% of family, 39% of religious institutions, 31% of peers, 28% of media and 20% of health institutions). Similarly, about 225 (23.6%) of the study participants had exposure to people with similar problems as described in the case vignette (60% in religious institutions, 32% someone close to them, 31.6% their neighbors and 15.6% on the street).

### Labeling and perception regarding the causes of schizophrenia

A Majority (73.5%) of the participants label the case vignette description as “Ebid” (a local language analogous to “madness” in English), 130(13.6%) as substance abuse and 81(8.5%) as malaria. The traditional perceptions of the causes of schizophrenia was73.7% (95% CI: 70.7, 76.5%). “Evil spirit” and punishment for sins/wrongdoings were attributed as causes of schizophrenia by 680 (71.4%) and 600 (62.8%) of respondents, respectively **(**Table 2**).**

**Table 2:** Perception regarding the causes of schizophrenia among Feresbet district residents, December 1st, 2016 to February 1st, 2017 (n = 952)

Possible Perceived cause	Yes		No	
	Frequency	Percentage	Frequency	Percentage
Work load	169	17.8	783	82.2
Financial difficulties	111	11.7	841	88.3
Bad methods of upbraiding	90	9.5	862	90.5
Problems in study	71	7.5	881	92.5
Illness/or death of family member	109	11.4	736	77.3
Conflict among relatives	58	6.1	894	93.9
Social environment	45	4.7	907	95.3
Other social causes	27	2.8	925	97.2
Cultural influence	37	3.9	915	96.1
Personality problem	53	5.6	899	94.4
Too much thinking	501	52.6	451	47.4
Alcohol/ drug misuse	271	28.5	682	71.5
Low educational level	19	2.00	933	98.00
Conflict in non-family relation	47	4.9	905	95.1
Marital quarrels	78	8.2	874	91.8
Conflict with spouse	77	8.1	875	91.9
Conflict with in law	16	1.7	936	98.3
Conflict with other relatives	24	2.5	928	97.5
Heredity factor	86	9.00	866	91.00
Stress	186	19.5	766	80.5
Fatigue	20	2.1	932	97.9
Other physical illness	30	3.2	922	96.8
Head injury	144	15.1	808	84.9
biological deficiency	21	2.2	931	97.8
Menses	4	0.4	948	99.6
Fate	51	5.4	901	94.6
Attention seeking behavior	42	4.4	910	95.6
Evil sprit	680	71.4	272	28.6
Evil eye	30	3.2	922	96.8
Goodwill	516	54.2	436	45.8
Witchcraft	580	60.9	372	39.1
Magic	59	6.2	893	93.8
Curse	294	3.9	658	96.1
Punishment for sins	600	62.8	352	37.2
Others (failure in love)	5	0.45	947	99.55

### Factors associated with traditional perception regarding the causes of schizophrenia

In the multivariable analysis, female sex, older age, single in marital status, lower educational level, and the joint family system were identified as statistically significant predictors for traditional perception regarding the cause of schizophrenia (Table 3).

**Table 3:** Factors associated with traditional perception regarding the causes of schizophrenia among Feresbet district residents, December 1st, 2016 to February 1st, 2017(n = 952)

Variable	Categories	Perceived cause		COR with 95% CI	AOR with 95% CI
		Traditional	Bio-psychosocial		
Age in years	18–24	170	107	1.00	1.00
	25–44	244	99	1.55 (1.10,2.17)	1.61(1.07,2.43)*
	45–64	156	30	3.27 (2.06,5.18)	3.66(2.13,6.30)***
	> = 65	132	14	5.93(3.25,10.83)	5.99(3.05,11.77)***
Sex	Male	271	136	1.00	1.00
	Female	431	114	1.89 (1.41,2.54)	1.75 (1.24,2.48)**
Marital status	Married	207	120	1.00	1.00
	Single	278	69	2.33 (1.65,3.30)	2.94 (1.89,4.58)***
	Separated	81	12	3.91 (2.05,7.47)	4.59 (2.15,9.80)***
	Divorced	58	21	1.60 (0.92,2.76)	1.58 (0.84,3.00)
	Widowed	78	28	1.61 (0.99,2.62)	1.18 (0.66,2.10)
Educational level	Unable to read& write	149	15	6.00(3.31,11.10)	4.00 (2.06,7.78)***
	Able to read &write	122	17	4.36 (2.44,7.78)	2.56 (1.30,5.04)**
	Primary	143	42	2.06 (1.32,3.22)	1.19 (0.70,2.03)
	Secondary	158	97	0.99 (0.67,1.44)	0.69 (0.43,1.09)
	Diploma and above	130	79	1.00	1.00
Occupational status	Government employed	117	98	1.00	1.00
	Unemployed	138	26	4.44 (2.70,7.31)	5.25(2.95,9.32)***
	Private business	151	33	3.83 (2.41,6.08)	4.04 (2.33,7.00)***
	Daily laborer	38	11	2.89 (1.40,5.96)	2.29 (0.97,5.37)
	Homemakers	169	25	5.66 (3.44,9.32)	4.82 (2.75,8.43)***
	Student	63	47	1.12 (0.70,1.78)	0.72 (0.40,1.31)
	Others^a^	26	10	2.17 (1.00,4.73)	1.08 (0.43,2.69)
Family system	Nuclear	545	226	1.00	1.00
	Extended	157	24	2.71(1.71,4.28)	2.18 (1.28,3.70)*
Exposure to mentally ill person/s	Yes	175	50	1.32(0.93,1.89)	0.89 (0.58,1.36)
	No	527	200	1.00	1.00

*P-value< 0.05, **p < 0.01, ***p < 0.001

^a^others = local beer making, house servant etc

## Discussion

The result of the current study showed that there were different traditions/supernatural perceptions and beliefs shared by Feresbet district residents regarding the causes of schizophrenia. Accordingly, the study found that 73.7% (95% CI: (70.7, 76.5%)) of respondents perceived the causes of schizophrenia as it is due to traditional reason. This finding was in line with a study done in Ghana (72.8%) [12]. Another qualitative study in southern Ethiopia is also supporting this finding (16). However, the result of the current was lower than the results from Pakistan (89.9%) [11] and Nigeria (96.8%) [23]. This difference might be explained by the social, cultural and religious difference of the study participants.

On the other hand, the finding of the current study showed a more traditional perception as compared to a study in Bali (65%) [20]. This difference might be due to the socio-cultural difference of the respondents and the difference in the data collection tools. The current study used CMQS and the later study used the Causal Belief Questioner (CBQ). The CMQS has more detail options which may overestimate possible perceived causes of schizophrenia than CBQ. The odds of perceiving schizophrenia as it is caused by traditional reason was 5.9 times higher among individuals with age of 65 and above as compared to age range of 18–24 years. This result is supported by studies done in Ethiopia, and Pakistan [21–23]. This might be explained due to the fact that older people might not have access for accurate sources of information about the causes of schizophrenia, and may have lower educational levels in the study area.

The odds of having traditional perception as the causes of schizophrenia among females were 1.7 times higher than men. But this is in contrast to a survey conducted by World Psychiatric Association which stated that women were more likely to understand schizophrenia as bio-psychosocial cause than men [22]. This difference might be explained by the fact that most females in our study area are at a lower educational level and house makers that may limit their exposure to different media and experience sharing opportunities [24].

The odds of sharing traditional perception as the causes of schizophrenia among single individuals were 2.9 higher as compared to married individuals. This finding is supported by other similar studies of China [13] and Pakistan [11]. This might be due to lack of access to domain of discussion and experience sharing among single individuals.

Similarly, the odds of having traditional perception regarding the causes of schizophrenia among individuals who were unable to read and write were 4 times higher than those with diploma and above. This is supported by different studies in Ethiopia [20], Pakistan [11] and Nigeria [21]. This might be due to the shortage opportunity to read and access media for those who unable to read and write.

Finally, respondents who are living in an extended family system were 2.1 times higher to have traditional perceptions about the causes of schizophrenia as compared to respondents living in a nuclear family system. This idea is supported by other similar studies done in Pakistan and South Africa [11, 25]. The possible reason might be due to the fact that people living in the extended family system can share some traditional perception from their ancestors /elder family members in their home.

## Conclusions

The traditional perception regarding the cause of schizophrenia was found to be higher than bio-psychosocial view. This demonstrates a need for sessions to individuals about the cause of schizophrenia. Female sex, no formal education, age ≥ 25 years, living in extended families and unemployment had statistically significant association with the traditional perception regarding the causes of schizophrenia. Therefore, giving special attention to females, uneducated and unemployed individual is crucial.

## Additional file

Additional file 1: Case vignetee description of schizophrenia. (PDF 186 kb)

## Abbreviations

AMSH: Amanuel mental specialized hospital
AOR: adjusted odd ratio
CI: Confidence interval
CMQS: Causal model questionnaire for schizophrenia
COR: Crude odd ratio
DSM: Diagnostic statistical manual
OR: Odds ratio
SD: standard deviation
SPSS: Statistical package for social science
WHO: World health organization
