# Supplementary material for: Perception regarding the causes of schizophrenia and associated factors among Feresbet district residents: a community based study
Source: BMC Public Health. 2019 Mar 25;19:338. doi: 10.1186/s12889-019-6678-4 (PMC6434636; doi:10.1186/s12889-019-6678-4)
Supplement: Supplementary file 1 — Case vignetee description of schizophrenia. (PDF 186 kb) [file 12889_2019_6678_MOESM1_ESM.pdf]

Case vignette

A 24 years old single girl has been acting strangely for the past seven months. She prefers to stay in isolation and has cut herself off from her close friends. She has a problem of keeping self hygiene and talks alone with some strange speech. She has even damaged materials available around her. When asked why she was doing so, she replied by saying that an external voice orders her to destroy. She also said that she is being chased by intelligence agencies and thinks that the news on the Television and radio are related to her.
